# Simulation and Optimization of FAPbI_3_ Perovskite Solar Cells with a BaTiO_3_ Layer for Efficiency Enhancement

**DOI:** 10.3390/ma15207310

**Published:** 2022-10-19

**Authors:** Denis Stanić, Vedran Kojić, Mario Bohač, Tihana Čižmar, Krunoslav Juraić, Thomas Rath, Andreja Gajović

**Affiliations:** 1 Department of Physics, University of Osijek, Trg Ljudevita Gaja 6, 31000 Osijek, Croatia; 2 Ruđer Bošković Institute, Bijenička Cesta 54, 10000 Zagreb, Croatia; 3 Institute for Chemistry and Technology of Materials, NAWI Graz, Graz University of Technology, Stremayrgasse 9, 8010 Graz, Austria

**Keywords:** perovskite solar cell, SCAPS-1D, optimization, simulation, power conversion efficiency, BaTiO_3_

## Abstract

Since the addition of BaTiO_3_ in perovskite solar cells (PSCs) provides a more energetically favorable transport route for electrons, resulting in more efficient charge separation and electron extraction, in this work we experimentally prepared such a PSC and used a modeling approach to point out which simulation parameters have an influence on PSC characteristics and how they can be improved. We added a layer of BaTiO_3_ onto the TiO_2_ electron transport layer and prepared a PSC, which had an FTO/TiO_2_/BaTiO_3_/FAPbI_3_/spiro-OMeTAD/Au architecture with a power conversion efficiency (*PCE*) of 11%. Further, we used the simulation program SCAPS-1D to investigate and optimize the device parameters (thickness of the BaTiO_3_ and absorber layers, doping, and defect concentration) resulting in devices with *PCE*s reaching up to 15%, and even up to 20% if we assume an ideal structure with no interlayer defects. Our experimental findings and simulations in this paper highlight the promising interplay of multilayer TiO_2_/BaTiO_3_ ETLs for potential future applications in PSCs.

## 1. Introduction

Within the past decade, organometal halide perovskites have become the forerunning thin film material for the development of next-generation solar cells. Synthesized by chemical solution processing, the cubic perovskite structure shows remarkable properties as an active layer in solar cell devices. Formamidinium-based lead perovskites have been widely used for several years, characterized by an optimal optical band gap of 1.47 eV [1], a high charge carrier mobility for polycrystalline films measuring 2.5 cm^2^ V^−1^ s^−1^, as well as carrier diffusion lengths with values of 1 µm [2]. However, obtaining solar cells with high efficiency still depends highly on the experience and craft of the research team. It is therefore difficult for new groups to comprehend all the potential factors that can influence the quality of perovskite films and solar cells. In that sense, numerical simulation can be a very useful tool for studying solar cell performance. One of these tools is the SCAPS 1D software (version 3.3.09), which has been shown to be useful for investigating various solar cell parameters such as device thickness, resistance, and temperature [3], the theoretical impact of novel hole transport layers [4], and multiple terminal tandem perovskite devices [5], or even estimating the potential solar cell devices comprising chalcogenide-based perovskite active layers [6].

Regarding the use of BaTiO_3_ in perovskite solar cells, its most prevalent application is as an intermediate layer between the electron transport layer (ETL), e.g., TiO_2_, and the perovskite active layer. As reported in the current literature, by adding the BaTiO_3_ interlayer, depending on the layer parameters, e.g., thickness, there is an increase in efficiencies in regards to the un-modified perovskite cells. This is probably due to several phenomena. The first group, to our knowledge, to investigate this complex ETL was Okamoto et al. in 2016 [7]. They showed that the crystal size of the perovskite increases when prepared on BaTiO_3_-modified TiO_2_, which leads to an increase in light absorption and a decrease in the number of grain boundaries that serve as trap sites for photogenerated charges. Several papers were later published [8,9,10] indicating that the addition of BaTiO_3_ onto TiO_2_ reduces charge recombination and increases charge separation due to more favorable energy alignments of conduction and valence bands of the different layers.

In a recent research study [11], we synthesized perovskite solar cells (PSCs) with bare TiO_2,_ which acts as an electron transport layer with a power conversion efficiency (*PCE*) of 7%, and showed that it can theoretically reach up to 15%. In that study [11], we discussed in detail the experimental approach for the advancement of the parameters that could lead to an improvement in the *PCE*. Since we found that the interface between layers considerably influences the PSC, we continue our study in the direction of interface engineering.

In this paper, in order to increase the *PCE* of a FAPbI_3_-based solar cell, we added a layer of BaTiO_3_ between the perovskite absorber and the TiO_2_ electron transport layer. This layer should affect the local electric field, enhance the extraction of electrons from the perovskite layer, and thus increase the current and performance of the PSC. Theoretical simulations were performed using SCAPS-1D software, which is primarily made for the simulation of thin film solar cells but is also widely used for modeling PSCs [12,13,14]. Using SCAPS-1D, we modeled a PSC with an additional BaTiO_3_ layer and in the first calculations, we found a *PCE* of 10%, which is 3% more efficient compared to the PSC we modeled without the BaTiO_3_ layer in our previous work [11]. According to this result, we synthesized a new PSC with a BaTiO_3_ layer between the perovskite and TiO_2_ layer and we produced a device with an experimental *PCE* of 11%, which is even 1% higher than the previous simulation result. Therefore, SCAPS was first used to fit the experimental curve, and we obtained a simulated *PCE* of 10.7%. Then, further optimization of the PSC parameters that can affect its performance (thickness of the BaTiO_3_ and absorber layers, doping, and defect concentration) was performed, and the result indicated that the *PCE* could reach a value of 14.71%. If we further assumed an ideal structure of the PSC with a low impact of interlayer resistivity, our device could theoretically reach over 20% *PCE*. 

## 2. Materials and Methods

### 2.1. Preparation and Characterization of Perovskite Solar Cells

The prepared solar cells were comprised of a multilayer n-i-p architecture. On top of the glass/FTO substrate, a planar TiO_2_ thin film was deposited using DC magnetron sputtering with an added spin-coated mesoporous BaTiO_3_ layer, which together served as an electron transport layer (ETL). On top of the ETL, a formamidinium lead iodide perovskite active layer was deposited using spin-coating. The hole transport layer (HTL), spiro-OMeTAD (≥99%, Merk, Darmstadt, Germany), was also spin-coated on top of the active layer. Finally, gold contacts were deposited using thermal evaporation. The perovskite solution preparation, active layer, HTL layer, and gold contact deposition were all conducted inside a nitrogen-filled glove-box (MBraun Labmaster system, Garching, Germany).

Prior to cleaning and magnetron deposition, the 15 × 15 mm FTO substrates (7 Ω/sq, Sigma Aldrich, St. Louis, MO, USA) were patterned using a small amount of Zn powder (p.a., T.T.T. Ltd., Sveta Nedelja, Croatia) and 2.5 M HCl (37%, p.a., Val-de-Reuil Carlo Erba Reagens, France). The middle FTO strip (9 × 15 mm) was covered and preserved prior to patterning using scotch tape, and cut to match the desired dimensions. The Zn powder was applied onto the substrate and HCl was added dropwise until the powder was saturated and started to react violently. This reaction was conducted in a fume hood and lasted for about 1–2 min until the reaction stopped. The patterning resulted in a substrate with two glass strips and an FTO strip in between, serving as the transparent contact of the cell. After patterning, the scotch tape was removed and the substrates were rinsed with water. The substrates were then cleaned in an ultrasonic bath submerged in acetone (99.9% Gram-mol Ltd., Zagreb, Croatia), then isopropanol (99.9% Gram-mol Ltd., Zagreb, Croatia) for 10 min each. After sonication, the substrates were rinsed in water and ethanol (97% Gram-mol Ltd., Zagreb, Croatia), dried in a nitrogen stream, and finally cleaned in an L2002A2 UV Ozone Cleaner (Ossila, Sheffield, UK) for 10 min to remove any residual organic compounds.

The compact TiO_2_ (c-TiO_2_) thin films were deposited using reactive DC magnetron sputtering. The sputtering process was conducted using a 2’’ diameter Ti target (99.995% Kurt J. Lesker, Saint Leonards-on-sea, UK) without additional heating at a set DC power of 100 W. Prior to the deposition, the magnetron chamber was evacuated to a high vacuum base pressure of 1∙10^−6^ mbar. The working gas that was introduced for the sputtering procedure was a mixture of argon and oxygen (p(O_2_)/p(Ar) = 0.2), which increased the pressure to 5 mTorr. The deposition lasted for 45 min, and afterward the samples were annealed at 450 °C for 2 h to induce crystallinity (anatase TiO_2_).

The mesoporous BaTiO_3_ (m-BaTiO_3_) thin films were prepared on top of the magnetron sputtered TiO_2_ thin films by spin-coating (H6-23 Spin Coater, Laurell Technologies Corporation, North Wales, PA, USA) a diluted BaTiO_3_ suspension at 4000 rpm for 30 s followed by thermal annealing at 450 °C for 2 h. The BaTiO_3_ stock suspension was prepared by mixing 170 mg of cubic BaTiO_3_ nanopowder (≥99%, Sigma Aldrich, St. Louis, MO, USA, particulates <100 nm) with 85 mg of dioctyl sulfosuccinate sodium salt (96%, Alfa Aesar, Kandel, Germany) and 850 mg of α-terpineol (90%, Sigma Aldrich, St. Louis, MO, USA). This stock solution had a consistency of a paste and was mixed for 24 h and intermittently sonicated to ensure good homogeneity. To prepare the suspension for spin-coating, 150 mg of the stock suspension was diluted in 1 mL of ethanol (99.9%, Gram-mol Ltd., Zagreb, Croatia) and was also mixed for 2 h and intermittently sonicated.

To prepare the perovskite thin films, 461 mg of PbI_2_ (99%, Sigma Aldrich, St. Louis, MO, USA) and 172 mg of formamidinium iodide (FAI, ≥99%, Sigma Aldrich, St. Louis, MO, USA) were dissolved in 1 mL of dimethylformamide/dimethyl sulfoxide (both ≥99%, Merck, Darmstadt, Germany) mixture (V(DMF):V(DMSO) = 4:1) in order to prepare a 1 mmol/mL solution of the precursor. Perovskite films were prepared inside a nitrogen-filled glovebox by spin coating 50 µL of the perovskite precursor on top of the m-BaTiO_3_ films. The spin-coating of the perovskite layer was performed using the two-step procedure: for the first step, the solution was spin-coated for 10 s at a speed of 1000 rpm and an acceleration of 200 rpm/s, after which the speed was increased to 6000 rpm (acceleration: 2000 rpm/s) for the next 20 s. During the last 10 s of perovskite spin-coating, 75 µL of chlorobenzene (≥99% Merck, Darmstadt, Germany) was dripped onto the rotating substrate. The as prepared substrates were annealed on a hotplate at 150 °C for 45 min.

The spiro-OMeTAD solution was prepared by dissolving 50 mg of spiro-OMeTAD in 498 μL of chlorobenzene and adding 18 μL of 4-tert-butylpyridine (tBP, 99% Sigma Aldrich, St. Louis, MO, USA), 10 μL of lithium bis(trifluoromethanesulfonyl)imide (LiTFSI, 99% Sigma Aldrich, St. Louis, MO, USA) stock solution, and 4 μL of tris(2-(1H-pyrazol-1-yl)-4-tert-butylpyridine)cobalt(III) tri[bis(trifluoromethane)sulfonimide] (FK209, 99% Sigma Aldrich, St. Louis, MO, USA) stock solution. The stock solution molar concentrations of LiTFSI and FK209 were 1.8 mmol/mL and 0.25 mmol/mL in acetonitrile (p.a. Merck, Darmstadt, Germany), respectively. Before spin-coating the spiro-OMeTAD layer, the spin-coater was dried with a continuous flow of nitrogen in order to remove residual DMF and DMSO vapors left from the preparation of perovskite thin layers. Afterward, 50 µL of the spiro-OMeTAD solution was spin-coated on the perovskite film (4000 rpm, 1000 rpm/s) for 10 s. The substrates were left resting overnight in dark and dry air. For the final step, 100 nm of gold contacts were deposited on the substrates by thermal evaporation.

The J-V measurements were conducted in a nitrogen-filled glove box using a Keithley 2400 (Tektronix Ltd., Oldbury, UK) source meter and a DLH400D lamp (Dedo Weigert Film GmbH, Munich, Germany) calibrated to 100 mW/cm^2^ using a reference silicon cell. The area of one cell was 0.09 cm^2^ and the potential sweep was performed at a scan speed of 20 mV/s in the range between −50 and 1100 mV. For this paper, a total of 12 solar cells were prepared (six cells at a time) and characterized. The successful cells had similar characteristics so one of them was used as the starting point for the modeling and optimization of the photovoltaic system. 

**Figure 1:**
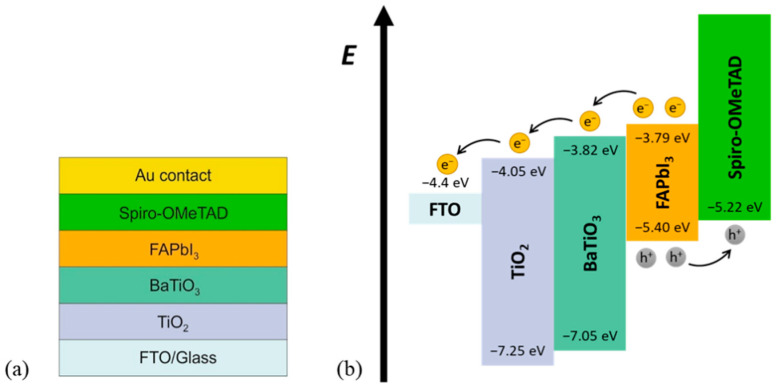
(**a**) The perovskite solar cell layer structure and (**b**) PSC band diagram [8,15].

The layer structure of the prepared PSCs is illustrated in Figure 1a, while the energy band diagram of the structure is shown in Figure 1b. This diagram shows a good matching of ETL and HTL with the absorption layer (FAPbI_3_), which allows efficient extraction of electrons and holes, respectively.

### 2.2. Numerical Simulation

The simulation was performed by using the solar cell capacitance simulator SCAPS-1D, which is based on solving one-dimensional continuity and Poisson equations [16]. It can simulate solar cell structures and calculate their basic characteristics, such as band diagrams, external quantum efficiency, generation and recombination profiles, cell current densities, *J–V* characteristics including open-circuit voltages (*Voc*), short-circuit currents (*Jsc*), fill factor (*FF*), and power conversion efficiency (*PCE*). For SCAPS simulations, the input parameters are taken from the literature [2,11,17,18,19,20,21,22,23,24,25,26,27] and our experimental results, which are listed in Table 1. The interface defects at ETL/absorber and absorber/HTL interfaces are considered neutral and single. The work function of FTO and back gold contact are set to 4.4 and 5.1 eV, respectively. All the simulations are performed at a working temperature of 300 K using a series resistance of 1 Ω and under standard AM1.5G illumination.

## 3. Results and Discussion

**Figure 2:**
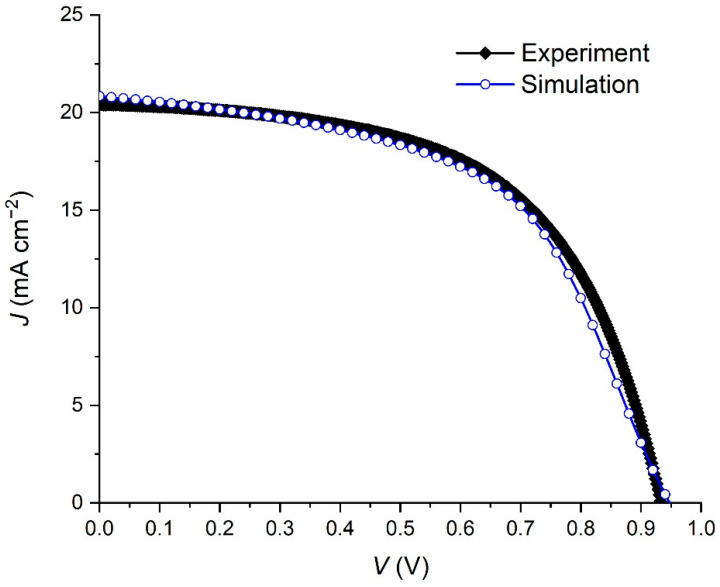
Current density–voltage characteristics under illumination of the experimental and simulated PSC with the device architecture FTO/TiO_2_/BaTiO_3_/FAPbI_3_/spiro-OMeTAD/Au.

Using the data from Table 1, we have simulated the current density–voltage (*J*–*V*) characteristic of the prepared PSC. The results are shown in Figure 2, which compares the experimental and simulated *J*–*V* characteristics. In addition, the comparison between the main solar cell parameters is shown in Table 2. A good match between experimental and simulation results is observed, which is a validation of our simulation model.

### 3.1. The Impact of the BaTiO_3_ Layer Thickness on the Performance of the PSC

There are several phenomena reported that improve the efficiencies of a PSC when a BaTiO_3_ interlayer is added. When the perovskite layer is prepared on BaTiO_3_-modified TiO_2_, its crystal size increases leading to higher light absorption and photogeneration of charged pairs [7]. At the same time, the number of grain boundaries, which serve as trap sites for photogenerated charges, decreases which lowers the recombination rate. Both of these effects enhance the efficiency of PSC. Furthermore, adding BaTiO_3_ onto TiO_2_ causes more favorable energy alignments of conduction and valence bands (Figure 1b) which increases charge separation and reduces charge recombination leading again to enhanced efficiency of the PSC. Recently, Zhang et al. [28] used ferroelectric properties of BaTiO_3_ material and increased the inner electric field of the PSC and the width of the depletion layer. This can lead to enhanced separation of charges and better transport of the carriers, which again raises the efficiency of PSC. On the other hand, it is difficult to synthesize a transparent, defect-free, and homogeneous thin film of BaTiO_3_ at low temperatures (since high temperatures can result in the TiO_2_ layer forming an undesirable rutile phase). This may induce a crystallization defect of the absorber layer which inhibits the expected efficient charge separation and transport. The interplay of these factors defines the net efficiency of the PSC.

**Figure 3:**
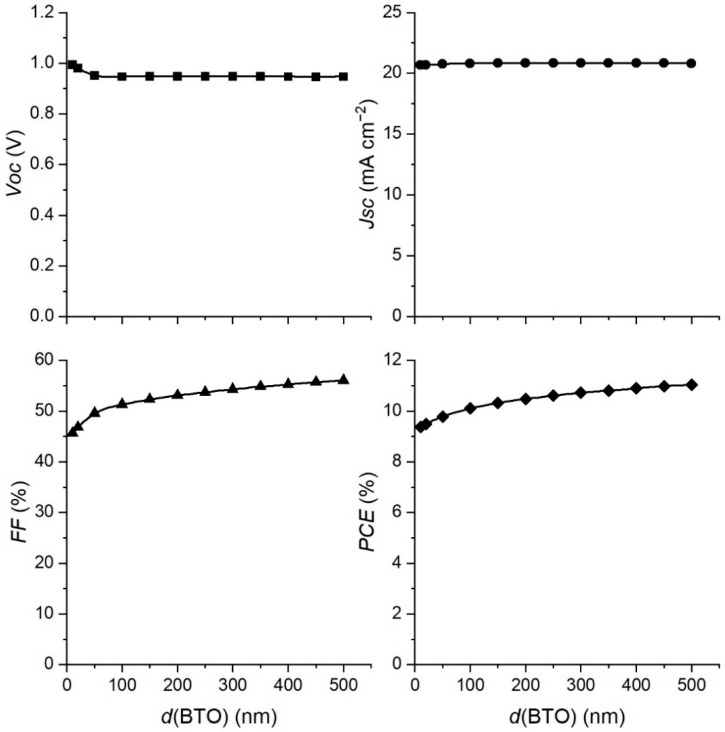
Variation of PSC parameters with the changing BaTiO_3_ layer thickness.

Since we added the BaTiO_3_ layer into our previous PSC structure [11] to improve its performance, the influence of the BaTiO_3_ layer thickness on the performance of the PSC was studied in order to find its optimal thickness. We simulated the performance in a range of thickness of the BaTiO_3_ layer from 10 to 500 nm, and the results are presented in Figure 3.

The open circuit voltage *Voc* and short circuit current *Jsc* stay almost constant with an increase in the thickness of the BaTiO_3_ layer. The addition of the BaTiO_3_ layer onto TiO_2_ causes more favorable energy alignments of conduction and valence bands. Once this alignment is fully developed, increasing the thickness of the BaTiO_3_ layer will not change it, and therefore *Voc* will stay constant. The charge separation will not be changed and *Jsc* will also stay constant. Thus, the only parameter that can influence the *PCE* is the *FF*. For this reason, the fill factor *FF* and the efficiency of the solar cell show the same saturation behavior, with the *PCE* rising from 9.4 to 11%. Since our experimentally prepared PSC device had a BaTiO_3_ layer with a thickness of 300 nm, and the *PCE* calculated from the fitting curve was 10.7% (pretty near the experimental value of 11%), we decided to take this thickness as an optimal value for further simulations. The experimental PSC with a thicker BaTiO_3_ layer would improve the device performance by only 0.3%, but would almost double the amount of BaTiO_3_ used in the production of the PSC.

### 3.2. Effect of Changing the Absorber Doping Concentration

**Figure 4:**
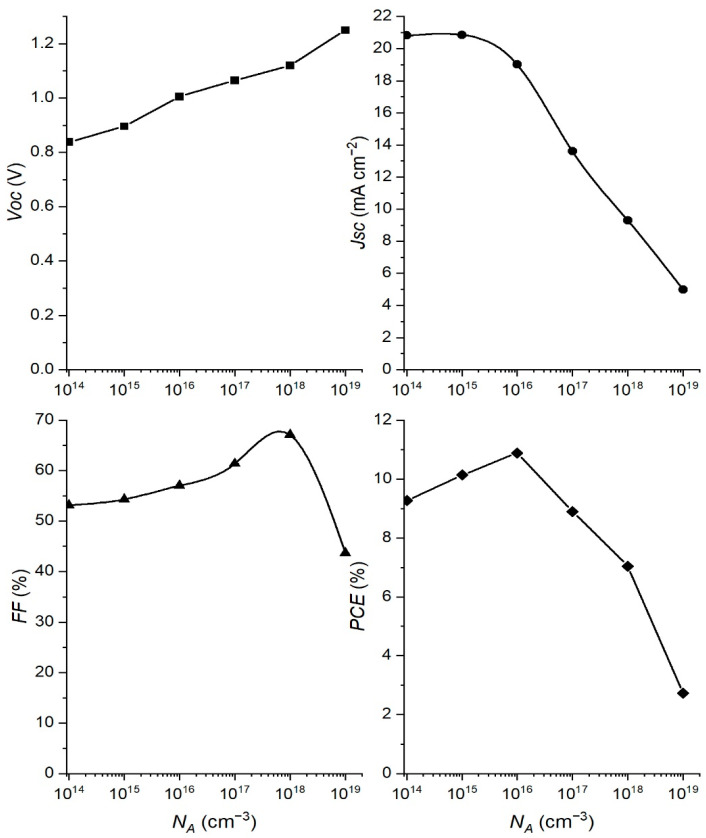
Variation of PSC parameters with the doping concentration *N_A_*.

Doping of the absorber layer is another important parameter that can affect the performance of the PSC, and we simulated the performance of PSC in the doping range of *N_A_* from 10^14^ cm^−3^ to 10^19^ cm^−3^. The rest of the parameters were kept constant and corresponded to the values indicated in Table 1. The simulation results of changing the PSC parameters with *N_A_* are shown in Figure 4. 

Increasing the doping concentration can increase the electric field at the perovskite interface and consequently enhance the process of charge separation. This can lead to an increase in *Voc*, as can be seen in Figure 4. On the other hand, increasing the *N_A_* concentration can cause an increase in the recombination rate that can negatively affect cell performance. This can be found in the behavior of the other PSC parameters (Figure 4), whose values decrease with increasing *N_A_*. 

Experimentally, when a perovskite thin film was synthesized by using a single cation (as is the case in this work where formamidinium as a single cation was used), the vacancies behave as dopants. The synthesis parameters can influence the nature of the thin film, so the crystallization of the active perovskite layer creates lattice strains, which can lead to vacancies or structural defects as is the case for polycrystalline thin films. The number of present vacancies and the defect concentration depends on the ratio of lead (II) iodide and formamidinium iodide in the precursor’s solution, as well as the choice of solvents [29] or the length of thermal annealing [30]. In this way, doping concentrations can be changed by changing the vacancy concentration without the necessity to add other cations.

**Figure 5:**
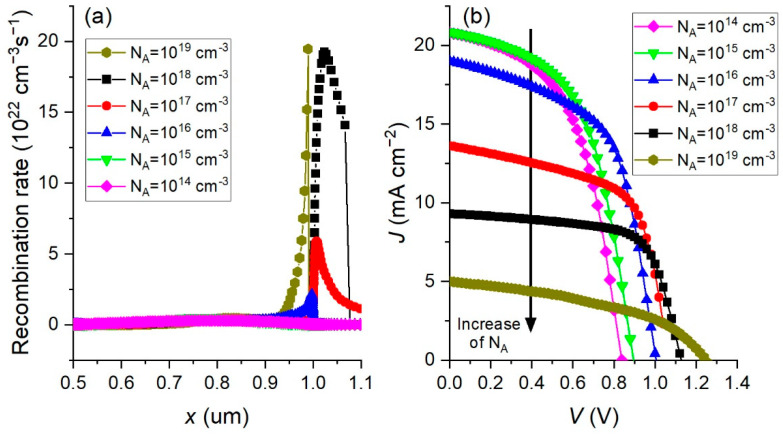
(**a**) Effect of changing the absorber layer’s doping concentration *N_A_* on recombination rates along the perovskite material. (**b**) Effect of increasing the doping concentration *N_A_* on *J*–*V* characteristics of the PSC device.

The influence of raising the doping concentration on the recombination inside the perovskite absorber material is presented in Figure 5a. The recombination rate is calculated by solving the Poisson and continuity equations in SCAPS-1D software where the doping concentration enters into the Poisson equation, as is described by Equations (1)–(5) in our previous work [11] and explained in Burgelman et al. [16]. Raising the doping concentration of *N_A_* increases the recombination rate that strongly affects the *J*-*V* curves, reducing the performance of the device (Figure 5b). 

Taking into account the *PCE* graph in Figure 4, the best performance of PSC is obtained if the concentration of *N_A_* = 10^16^ cm^−3^ is used as the optimum one for further simulation. Since the starting concentration of *N_A_* = 1.9·10^15^ cm^−3^, the result of the optimization tells us that we need to increase the absorber doping concentration in order to get the higher *PCE* of the cell.

### 3.3. Effect of Changing Absorber Layer Thickness

**Figure 6:**
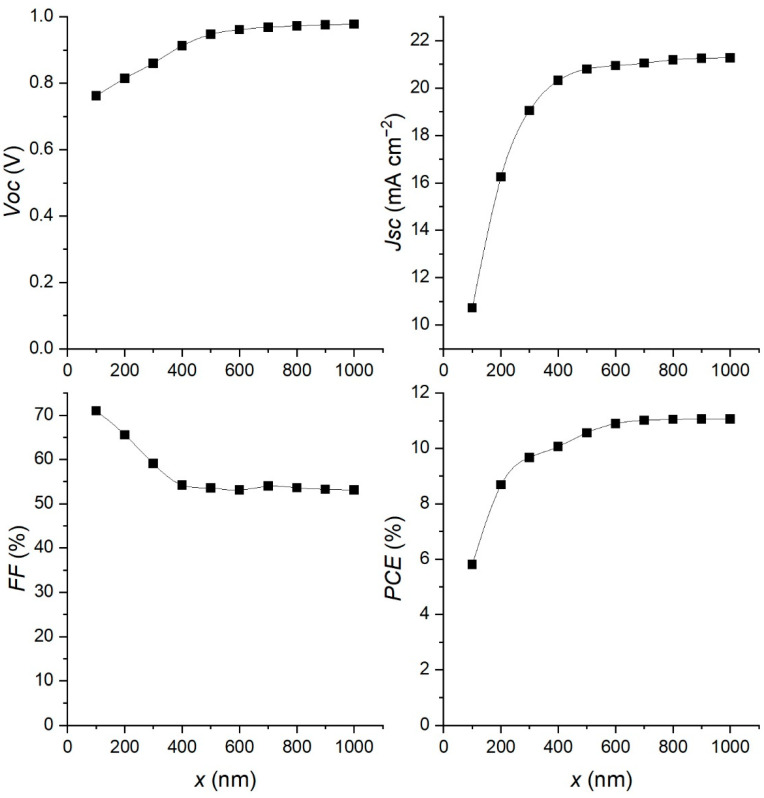
Impact of increasing absorber thickness on PSC device performance.

The thickness of the absorber layer is another parameter that can affect the behavior of the PSC. In order to study its influence on the cell performance, the thickness is changed in the interval from 100 nm to 1000 nm, and the results are shown in Figure 6.

Thinner absorber layers result in lower light absorption leading to lower values of photocurrent, and consequently lower values of *PCE*. As the thickness is increased, the amount of absorbed light is also increased, leading to higher values of current and *PCE*. However, it also increases the recombination rates in the bulk (depending on the diffusion length), saturating the *Jsc* and *PCE* values for absorber layer thicknesses higher than 600 nm. Thus, this value is chosen as an optimum for the thickness of the absorber layer. The *Voc* also shows saturation for the higher values of the absorber thickness, while the FF is decreasing to 53%.

### 3.4. Effect of Changing the Defect Concentration N_t_

In order to improve the PSC performance, the change in defect density was considered. Polycrystalline films tend to have a larger number of defects in comparison to monocrystalline films, and synthesis techniques such as crystal-oriented growth [31] can influence the concentration of present defects. The morphology and film quality of the perovskite layer have an important influence on the performance of the perovskite solar cell [32]. Poor quality and film coverage on mesoporous TiO_2_ have been shown to increase the charge recombination inside the active layer [33]. The recombination is explained by the increase in the defect density (*N_t_*), which can impact the *Voc* of the solar cell.

To study the influence of the defect density of the perovskite active layer on the cell performance, the Shockley–Read–Hall recombination model (SRH) was used. The neutral defects were set at the center of the band gap following the Gaussian distribution with the characteristic energy value of 0.1 eV, centered in the middle of the band gap. In the SRH recombination model, the recombination rate *R* is given by [34,35]:

(1) $$R=\frac{np-n_{i}^{2}}{\tau_{p}\left(n+N_{C}e^{\left(E_{C}-E_{t}\right)/kT}\right)+\tau_{n}\left(p+N_{V}e^{\left(E_{t}-E_{V}\right)/kT}\right)},$$

where *n* and *p* are the concentrations of the mobile electrons and holes, respectively. These concentrations can be found by solving the continuity and Poisson equations. At positive voltage values, where *qV >* 3 *kT*, the term $n_{i}^{2}$, which explains the thermal generation, can be neglected. *E_t_* represents the energy level of the trap defects, while *N_t_* is their concentration. *τ_n_* and *τ_p_* are the lifetimes of the electrons and the holes, respectively, and are given by the following equations:

(2) $$\tau_{n}=\frac{1}{\sigma_{n}v_{th}N_{t}},\;\;\;\;\tau_{p}=\frac{1}{\sigma_{p}v_{th}N_{t}},$$

where *σ_n_* and *σ_p_* are the capture cross-sections of the electrons and holes, respectively, and *v_th_* represents the thermal velocity. 

The diffusion length *l* of the carrier is given by the equation:

(3) $$l=\sqrt{D\tau },$$

where *D* is the diffusion coefficient defined by the equation:

(4) D=μkT/q,

where *μ* is the charge carrier mobility. According to Equation (2), when defect density decreases, the charge carrier lifetimes increase, leading to longer diffusion lengths (Equation (3)) and a lower recombination rate (Equation (1)). These are the main factors influencing the improvement of cell performance. 

**Figure 7:**
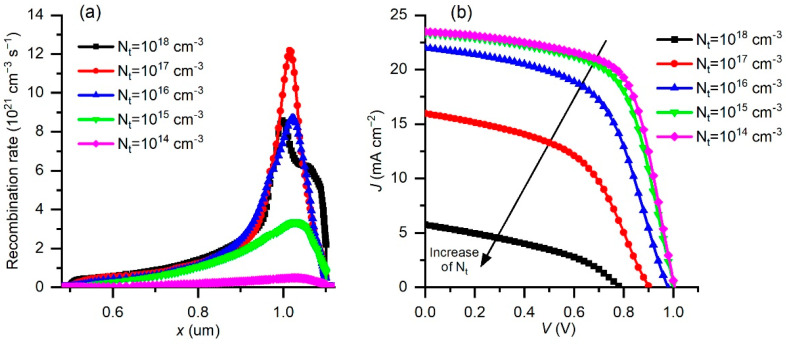
(**a**) Change in recombination rate as a function of depth in the absorber layer for different *N_t_* values. (**b**) *J–V* characteristics of simulated PSCs as a function of defect density *N_t_*.

The defect density *N_t_* was investigated as a parameter in the PSC performance, and the defect density values were changed from 10^14^ cm^−^^3^ to 10^18^ cm^−^^3^. The change in the recombination rate (*R*) (Equation (1)) is shown in Figure 7a. It is clear that the reduction in *N_t_* lowers the recombination rate and at the same time increases the diffusion length *l*.

**Figure 8:**
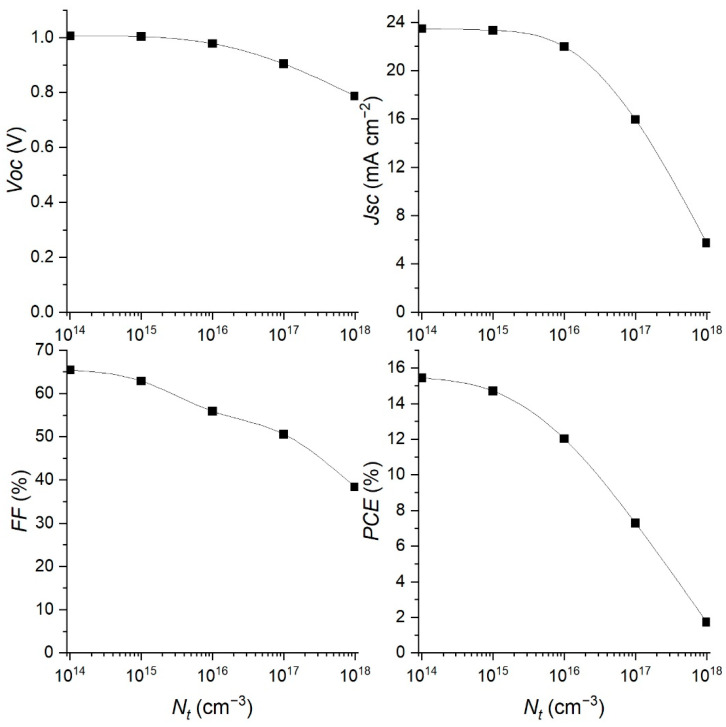
Effect of changing the *N_t_* concentration on the device parameters of the PSC.

Therefore, the reduction in defect density in the perovskite material can significantly improve the performance of the PSC, which is consistent with the simulation results shown in Figure 7b. The obtained simulated *J–V* characteristics reveal an improvement with the reduction in *N_t_*. The behavior of the PSC parameters with the change of the *N_t_* concentration is shown in Figure 8. The increase in *N_t_* reduces all the parameters, especially *Jsc* and *PCE* as soon as the *N_t_* crosses a value of 10^15^ cm^−3^. The recombination rate increases and reduces the charge concentration, which further decreases the current through the device. The charge separation is reduced too, so the *Voc* also drops with increasing *N_t_*, and the *FF* shows similar behavior.

**Figure 9:**
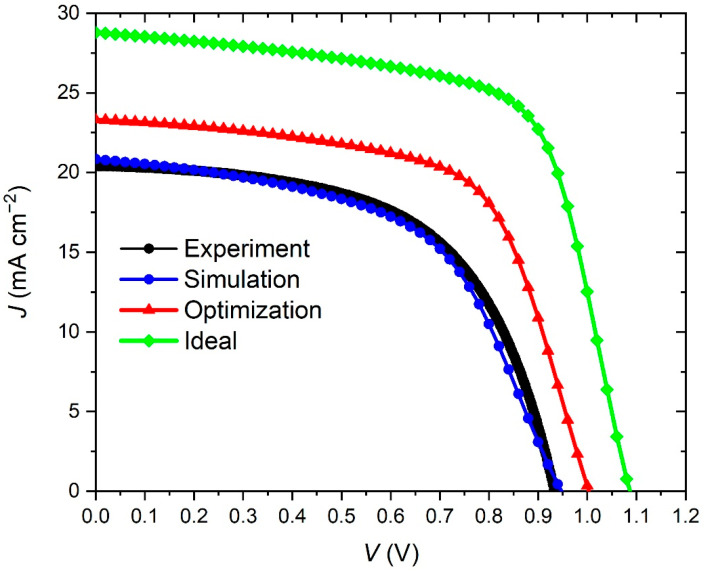
*J–V* characteristics: experimental, simulation (best fit), optimized, and ideal.

The best performance was obtained with the lowest defect density of 1·10^14^ cm^−^^3^, but it is very difficult to obtain such a low *N_t_* in experiments due to the polycrystalline nature of the perovskite films. Because of that, we set the optimized value of defect density at 1·10^15^ cm^−^^3^. As can be seen from Figure 8, this value of *N_t_*, gives the following PSC parameters: *Voc* = 1.00 V, *Jsc* = 23.32 mAcm^−2^, *FF* = 62.87% and *PCE* = 14.71%. The *J–V* curve with these optimized parameters is presented in Figure 9. Following the discussion in our previous research [11], if we can make the ideal structure of the PSC with low series resistivities and neglect the interlayer resistivities, we could have the ideal *J–V* characteristic (Figure 9). Its parameters are *Voc* = 1.09 V, *Jsc* = 28.79 mAcm^−2^, *FF* = 66.45%, *PCE* = 20.79%.

## 4. Conclusions

By adding a BaTiO_3_ layer in between the TiO_2_ ETL and the perovskite absorber layer, we improved the *PCE* of a FAPbI_3_-based solar cell from 7% to 11%. According to mentioned references, we consider that this improvement comes from influencing the electric field, which facilitated better charge separation and transport, and from a reduction in recombination processes. After using numerical simulation and optimization processes with SCAPS-1D, the theoretical *PCE* rises almost up to 15%. In an ideal device, the efficiencies can even reach 20%, verifying that the BaTiO_3_ layer can drastically improve the PSC performance and can be used in future research as a promising material for optoelectronic devices.

## Figures and Tables

**Table 1:** Basic input parameters of the materials used in the PSC.

Parameter	FTO [17,18]	TiO_2_ [11,18,19]	BaTiO_3_	FAPbI_3_ [17]	spiro-OMeTAD [17,18,21]
Thickness (nm)	300 *	100 *	300 *	550 *	500 *
Band gap (eV)	3.5	3.26	3.2 [22]	1.51	2.9
Electron affinity (eV)	4.0	4.2	3.8 [23]	4.0 [25]	2.2
Dielectric permittivity	9	9 [20]	2500 [24]	6.6 [26]	3
CB effective density of states (cm^−^^3^)	2·10^18^	2.2·10^18^	2.2·10^18^	1.2·10^19^ [27]	2.2·10^18^
VB effective density of states (cm^−^^3^)	1.8·10^19^	1.8·10^18^	1.8·10^18^	2.9·10^18^ [2]	1.8·10^18^
Thermal velocity of electrons (cm/s)	10^7^	10^7^	10^7^	10^7^	10^7^
Thermal velocity of holes (cm/s)	10^7^	10^7^	10^7^	10^7^	10^7^
Electron mobility (cm^2^/Vs)	20	20	20	2.7 [2]	10^−4^
Hole mobility (cm^2^/Vs)	10	10	10	1.8 [2]	10^−4^
Shallow donor density *N*_D_ (cm^−^^3^)	10^19^	5·10^16^	5·10^16^	0	0
Shallow acceptor density *N*_A_ (cm^−^^3^)	0	0	0	1.9·10^15^	10^18^
Defect density *N_t_* (cm^−^^3^)	10^15^	10^15^	10^15^	1.9·10^16^	10^15^

* Experimentally determined.

**Table 2:** Experimental and simulated parameters of the FAPbI_3_-based solar cell comprising a BaTiO_3_ interlayer.

Parameter	Experimental	Simulated
*Voc* (V)	0.93	0.94
*Jsc* (mA/cm^2^)	20.44	20.80
*FF* (%)	57.62	54.29
*PCE* (%)	11.00	10.72

## Data Availability

Not applicable.
